# Idiopathic pulmonary fibrosis in the United States: time to diagnosis and treatment

**DOI:** 10.1186/s12890-023-02565-7

**Published:** 2023-08-02

**Authors:** Michelle B. Herberts, Taylor T. Teague, Viengneesee Thao, Lindsey R. Sangaralingham, Henry J. Henk, Kevin T. Hovde, Timothy M. Dempsey, Andrew H. Limper

**Affiliations:** 1 Department of Pulmonary and Critical Care Medicine, Mayo Clinic, Gonda 18-South, 200 1st St SW, Rochester, MN 55905 USA; 2 Robert D. and Patricia E. Kern Center for the Science of Health Care Delivery, Mayo Clinic, Gonda 18-South, 200 1st St SW, Rochester, MN 55905 USA; 3 OptumLabs ®, 1 Main Street #10, Cambridge, MA 02142 USA; 4 David Grant Medical Center, US Air Force, Travis AFB, CA 94535 USA

**Keywords:** Idiopathic pulmonary fibrosis, Diagnosis, Treatment, Oxygen, Mortality

## Abstract

**Objective:**

Create a timeline of diagnosis and treatment for IPF in the US.

**Design, setting, and participants:**

A retrospective analysis was performed in collaboration with the OptumLabs Data Warehouse using an administrative claims database of Medicare Fee for Service beneficiaries. Adults 50 and over with IPF were included (2014 to 2019).

**Exposure:**

To focus on IPF, the following diagnoses were excluded: post-inflammatory fibrosis, hypersensitivity pneumonitis, rheumatoid arthritis, sarcoidosis, scleroderma, and connective tissue disease.

**Main outcomes and measures:**

Data were collected from periods prior, during, and following initial clinical diagnosis of IPF. This included prior respiratory diagnoses, number of respiratory-related hospitalizations, anti-fibrotic and oxygen use, and survival.

**Results:**

A total of 44,891 with IPF were identified. The most common diagnoses prior to diagnosis of IPF were upper respiratory infections (47%), acute bronchitis (13%), other respiratory disease (10%), chronic obstructive pulmonary disease and bronchiectasis (7%), and pneumonia (6%). The average time to a diagnosis of IPF was 2.7 years after initial respiratory diagnosis. Half of patients had two or more respiratory-related hospitalizations prior to IPF diagnosis. Also, 37% of patients were prescribed oxygen prior to diagnosis of IPF. These observations suggest delayed diagnosis. We also observed only 10.4% were treated with anti-fibrotics. Overall survival declined each year after diagnosis with median survival of 2.80 years.

**Conclusions and relevance:**

Our retrospective cohort demonstrates that IPF is often diagnosed late, usually preceded by other respiratory diagnoses and hospitalizations. Use of available therapies is low and outcomes remain poor.

**Supplementary Information:**

The online version contains supplementary material available at 10.1186/s12890-023-02565-7.

## Introduction

Idiopathic pulmonary fibrosis (IPF) is the most common type of fibrosing interstitial lung disease affecting an estimated 2.8–9.3 cases per 100,000 people per year [1]. Onset generally occurs in the 7th decade of life. Traditional risk factors include age, male gender, tobacco use history, and family history. The diagnosis of IPF can be challenging, as it requires the exclusion of other causes of fibrosing interstitial lung disease (ILD) such as hypersensitivity pneumonitis, exposures to various medications, radiation, and environmental exposures to inorganic dusts and organic agents associated with hypersensitivity reaction, as well as rheumatologic diseases [2].

Differentiating IPF from other causes of ILD is important as it can lead to different approaches to treatment and altered prognosis [3]. Treatment initiation with anti-fibrotic therapy has been shown to reduce short term mortality and hospitalizations but is unfortunately often initiated late in the disease course [4–6]. Unfortunately, the prognosis for IPF remains grim with survival ranging from 3 to 5 years after diagnosis [7]. Increased mortality is associated with markers of more severe disease including oxygen use, lower forced vital capacity (FVC) and reduced diffusing capacity for carbon monoxide (DLCO) [8, 9]. Since IPF is a diagnosis of exclusion, it may be challenging to definitively prove that some patients are suffering from IPF. This can lead to a delay in diagnosis and initiation of therapy. It has been proposed that late initiation of treatment may limit the overall benefit of these agents [10].

As it is widely held that patients with IPF experience diagnostic delays based on data gathered from academic centers, we sought to describe the timeline to diagnosis and initiation of treatment for patients with IPF using real world datasets. Our retrospective cohort analysis is unique because we used data from the Center for Medicare Services Fee for Service database, which consists of over 50,000 patients with IPF across the United States. Therefore, the current study aimed to investigate the identified patients’ initial respiratory diagnosis, subsequent time to clinical diagnosis of IPF, timing of oxygen initiation and prescription of anti-fibrotic agents. We also obtained information regarding respiratory-related hospitalizations and survival.

### Study design

To refine our IPF code identification strategy, an initial local cohort analysis of 200 consecutive patients coded for IPF were first analyzed by medical record review. This retrospective cohort study included patients > 18 years of age who were present within our institution’s electronic record system between 2011 and 2019. ICD 9 and 10 codes for IPF (516.3 and J84.122) were used to identify the validation study cohort. Each patient’s clinical history, laboratory results (including serologies for connective tissue disease and hypersensitivities), exposure history, CT scan pattern, lung biopsy results, and competing diagnoses were reviewed independently by three physicians (MH, TT, AL), and consensus diagnoses derived (Supplementary Figure S1). From this initial validation cohort, we identified several diagnostic codes for competing diagnoses (i.e. hypersensitivity pneumonitis, post-inflammatory fibrosis, etc.) that were excluded in order to focus more specifically on IPF in the current diagnostic timeline study. In particular, in our review of 200 consecutive Mayo Clinic cases, we did not find any patients that were coded as post-inflammatory pulmonary fibrosis, but in actuality were established with a final diagnosis of IPF.

Using this refined diagnostic code strategy, the current study was a retrospective cohort analysis using the Medicare Fee-for-Service (FFS) data. The database contains longitudinal health information on enrollees, representing a diverse mixture of ages, ethnicities, and geographical regions across the United States [11]. Since this study involved analysis of pre-existing, de-identified data, it was deemed to be exempt of human studies research by the Mayo Clinic Institutional Review Board and the National Institutes of Health.

### Study population

We included all adult patients 50 years or older who had their first coded clinical diagnosis of IPF between January 1, 2014, and December 31, 2019. We then constructed an incident cohort by requiring individuals to have five years of continuous enrollment prior to their IPF diagnosis. IPF was identified using the following International Classification of Diseases, Ninth Edition (ICD-9) diagnosis codes: 516.31; and International Classification of Diseases, 10th Edition (ICD-10) diagnosis codes: J84.112, as previously described [12]. Patients without a diagnosis of IPF were excluded from our cohort as were patients that had a coded diagnosis of post-inflammatory fibrosis, connective tissue disease-related ILD, rheumatoid arthritis-related ILD, sarcoidosis, scleroderma, nonspecific interstitial pneumonia, and chronic hypersensitivity pneumonitis. Excluded ICD codes are shown in Supplementary Table S1. These diseases were excluded after cohort validation determined that these diagnostic codes were often confounded with IPF.

### Outcomes and other covariates

Descriptive variables of interest at index IPF diagnosis included age, gender, race/ethnicity, region of residence, and history of smoking (ICD-9: 649.0X, 305.1, 989.84, V15.82 and ICD-10: F17.X, O00.33X, T65.2X, Z53.01, Z71.6, Z72.0, Z87.891 and procedure codes: 1034 F, 4000 F, 4001 F, 4004 F, 99,406, 99,407, C9801, C9802, D1320, G0357, G0376, G0436, G0437, G8402, G8453, G8455, G9276, G9458, G9792). We further evaluated respiratory-related diagnoses and hospitalizations at baseline and after IPF diagnosis. Respiratory-related hospitalizations were identified using AHRQ’s Clinical Classification Software [13]. We evaluated time to initiation of oxygen and anti-fibrotic therapy, and length of time patients received anti-fibrotic medications. Oxygen use was identified from the following codes: E0424, E0425, E0430, E0431, E0433-E0435, E0440-E0447, E0455, E1352-E1354, E1356-E1359, E1391, E1392. Anti-fibrotic use was defined as any patient with IPF who filled a prescription for either pirfenidone or nintedanib at any time. In addition, we assessed the relevant codes for lung transplantation (CPT: 32,851–32,854, S2060) after initial coded diagnosis of IPF. We also analyzed overall patient survival using two different time stamps: start date of oxygen use and date of discharge after initial respiratory-related hospitalization. Survival time was defined as the period from start date of interest to death. Patients were censored at their last enrollment date or at study end (December 2019). There are four main sources of mortality information in this dataset: (1) Social Security Administration Death Master; (2) electronic health records identifying deceased status; (3) death as a reason for disenrollment in the health insurance plan; and (4) death indicated in the inpatient discharge status [14].

### Statistical analysis

The data from this observational cohort analysis are reported using descriptive statistics including frequencies and percentages or average mean values. We used the Fisher’s exact test for multiple group comparisons [15]. In addition, Kaplan Meier survival estimates were performed for patients following the initiation of oxygen therapy or following initial hospitalization. Statistical analyses were conducted with SAS, version 9.4.

### Role of funding sources

The funding source for this study played no role in study design; collection, analysis, or interpretation of the data; writing of the report; and decision to submit this paper for publication.

## Results

**Fig. 1:**
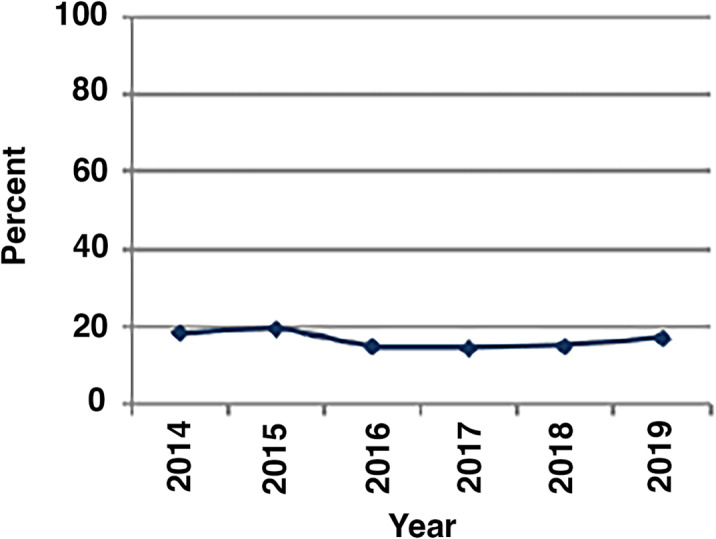
Year of Idiopathic Pulmonary Fibrosis Diagnosis. (A serial evaluation of the relative numbers of IPF cases diagnosed in each year of the study. Shown are the percentages of the total number of patients diagnosed with IPF in the cohort (total N = 44,891) that received their index diagnosis of IPF in each designated year)

Our initial local cohort analysis described above allowed us to exclude other competing diagnoses and focus more specifically on IPF (Supplementary Figure S1). Using this refined strategy, we identified 44,891 patients from the dataset that met criteria for inclusion in the study. The demographics of these patients at initial diagnosis are reported in Table 1. Most patients with IPF (65.3%) fell into the 75 to 85 age range. 20% of patients were in the 65–74 age range, 12.6% were in the 85 and over age group, and 1.6% were less than 65. In this cohort, IPF seemed to affect men and women equally, with 51% of patients being female. Most individuals were white, representing 92.8% of this population. The rest of the patient population included 2.9% Hispanic, 2% Black, and 2.4% “other”. Using the OptumLabs definition of geographic locations [4], our cohort included 41.5% of patients from the South, 24.9% from the Midwest, 17% from the West, and 16.2% from the Northeast. For comparison, in 2019 there were 4,517,459 total enrolled patients in the South, 1,490,787 in the Midwest, 2,370,456 in the West, and 1,295,254 in the Northeast. Prior to diagnosis, approximately 56.6% (N = 25,404) of patients had a history of tobacco use. The percentage of patients diagnosed with IPF each year was relatively stable over 2014 to 2019 with approximately 15–20% of the cohort being diagnosed each year (Fig. 1).

**Table 1:** Baseline Demographics of Patients with Idiopathic Pulmonary Fibrosis

	Treated with antifibrotics before coded IPF diagnosis (N = 1,205)	Treated with antifibrotics after coded IPF diagnosis (N = 3,444)	Untreated (N = 40,242)	Total (N = 44,891)	p value
**Age**					< 0.0001
Mean (SD)	77.4 (4.9)	76.9 (4.7)	79.0 (5.5)	78.8 (5.4)	
Median	77.0	77.0	80.0	80.0	
**Age Group**					< 0.001
Less than 65	14 (1.9)	35 (4.8)	676 (93.2)	725 (1.6)	
65–74	320 (3.5)	1,071 (11.6)	7,824 (84.9)	9,215 (20.5)	
75–84	794 (2.7)	2,212 (7.6)	26,288 (89.7)	29,294 (65.3)	
Over 85	77 (1.4)	126 (2.2)	5,454 (96.4)	5,657 (12.6)	
**Gender**					< 0.001
Female	472 (2.1)	1,257 (5.5)	21,046 (92.4)	22,775 (50.7)	
Male	733 (3.3)	2,187 (9.9)	19,196 (86.8)	22,116 (49.3)	
**Race/Ethnicity**					0.0039
White	1,139 (2.7)	3,248 (7.8)	37,274 (89.5)	41,661 (92.8)	
Hispanic	30 (2.3)	85 (6.6)	1,175 (91.1)	1,290 (2.9)	
Black	14 (1.6)	44 (4.9)	833 (93.5)	891 (2.0)	
Other	22 (2.1)	67 (6.4)	960 (91.5)	1,049 (2.3)	
**Census Region**					< 0.001
Midwest	311 (2.8)	857 (7.7)	9,999 (89.5)	11,167 (24.9)	
Northeast	186 (2.6)	511 (7.0)	6,597 (90.4)	7,294 (16.2)	
South	525 (2.8)	1,555 (8.3)	16,571 (88.8)	18,651 (41.5)	
West	181 (2.4)	520 (6.8)	6,948 (90.8)	7,649 (17.0)	
**History of Tobacco Use**	772 (64.1)	1,975 (57.3)	22,657 (56.3)	25,404 (56.6)	< 0.001

We found 98% (N = 44,096) of patients with IPF had other initial respiratory diagnosis in their baseline period before the index diagnosis of IPF. The most common respiratory-related diagnoses were upper respiratory infections (47%), acute bronchitis (13%), other upper respiratory disease (10%), chronic obstructive pulmonary disease and bronchiectasis (7%), and pneumonia (6%). Additional diagnoses can be found in Table 2. The average length of time from initial respiratory diagnosis to clinical diagnosis of IPF was 990 days or 2.7 years. About 70% of patients had two or more respiratory-related hospitalizations prior to their diagnosis of IPF (Table 3).

**Table 2:** Initial Respiratory Diagnosis Prior to Diagnosis of Idiopathic Pulmonary Fibrosis

Diagnosis^*^	ICD-10 CCS category	N	%
Other upper respiratory infections	126	20,560	46.63
Acute bronchitis	125	5,571	12.63
Other upper respiratory disease	134	4,407	9.99
Chronic obstructive pulmonary disease and/or bronchiectasis	127; RSP008	3,881	8.80
Pneumonia (except that caused by tuberculosis or sexually transmitted disease, e.g.chlamydial infection)	122	2,721	6.17
Pneumonia (except that caused by tuberculosis)	RSP002	1,115	2.53
Other specified upper respiratory infections	RSP006	1,036	2.35
Sinusitis	RSP001	760	1.72
Other lower respiratory disease	133	717	1.63
Other specified and unspecified lower respiratory disease	RSP016	669	1.52
Asthma	128; RSP009	624	1.42
	RSP008		
Pleurisy; pneumothorax; pulmonary collapse	130	505	1.15
Acute bronchitis	RSP005	375	0.85
Influenza	123; RSP003	253	0.57
Other specified and unspecified upper respiratory disease	RSP007	238	0.54
Bacterial infections	INF003	215	0.49
Allergic reactions	INJ031	137	0.31
Acute and chronic tonsillitis	124	66	0.15
Lung disease due to external agents	132	53	0.12
Respiratory failure; insufficiency; arrest (adult)	131	46	0.10
Pleurisy pleural effusion and pulmonary collapse	RSP011	43	0.10
Viral infection	INF008	37	0.08
Aspiration pneumonitis; food/vomitus	129	14	0.03
Respiratory failure; insufficiency; arrest	RSP012	12	0.03

^*^The diagnosis codes were derived from the ICD-10 CCS codes using software: https://hcup-us.ahrq.gov/toolssoftware/ccs10/CCSCategoryNames(FullLabels).pdf

**Table 3:** Number of Respiratory-Related Hospitalizations Before and After Idiopathic Pulmonary Fibrosis Diagnosis

	Hospitalized Before IPF Diagnosis N = 25,422	Hospitalized With or After IPF Diagnosis N = 26,029
**Number of hospitalizations**		
1	11,445 (45.02)	12,105 (46.51)
2	5,989 (23.56)	6,279 (24.12)
3	3,126 (12.30)	3,275 (12.58)
4	1,809 (7.12)	1,730 (6.65)
5+	3,053 (12.00)	2,640 (10.14)

The average time from initial respiratory-related hospitalization to diagnosis of IPF was 786 days or 2.2 years. Most patients with IPF were hospitalized with a respiratory-related diagnosis between baseline and follow-up (N = 35,243; 78.5%). A majority had a respiratory-related hospitalization prior to a diagnosis of IPF (N = 25,422, 56.6%) and many had additional hospitalizations after their diagnosis of IPF (N = 26,029, 71.6%). Furthermore, many patients had multiple hospitalizations. (Table 3)

Notably, we found that only 4,649 or 10.3% of patients with IPF were treated with an anti-fibrotic during their disease course (see Table 1 for breakdown by treatment). Of those treated, 1,205 (25.9%) were treated with an anti-fibrotic an average of 238 days or 7.9 months before their coded diagnosis of IPF whereas 3,444 (74.1%) were treated an average of 205 days or 6.8 months after their diagnosis of IPF. When we evaluated treatment by gender, age, race/ethnicity, and region, we found that 2.1% of females were treated with an antifibrotic before their IPF diagnosis, 5.5% treated after their IPF diagnosis, and that 92.4% did not receive any anti-fibrotic therapy. In contrast, 3% of males received antifibrotic treatment before their IPF diagnosis, 9.9% after their IPF diagnosis, and 86.8% did not receive any anti-fibrotic treatment. Overall, more males were treated with an antifibrotic than females (13.2 v 7.6%, p < 0.0001). It should be noted that the use of antifibrotics before the index diagnosis of IPF is most likely due to filing the prescription coding for antifibrotic, and administrative delay when the diagnosis of IPF was coded during the clinical claims billing, since the diagnosis of IPF is most often required for prescribing.

The use of antifibrotic agents also varied by the age of the individual (Table 1). The age group most commonly treated with antifibrotic agents was 65 to 74 years (6.7% in those less than 65 years, 15.1% in those aged 65–74, 10.3 in 75–84 years, and 3.6% in those 85 and older, p < 0.0001). Furthermore, differing rates of treatment with antifibrotics were also observed by race/ethnicity (Table 1, whites 10.5% vs. blacks 6.5% vs. Hispanics 8.9% vs. others at 8.5%, respectively, p < 0.0001). Treatment rates were relatively similar across all geographical regions. In total, patients with IPF were treated an average of 220 days or 7.3 months overall, and 24.6% (N = 1,146) of the patients did not refill their antifibrotics after their first prescription.

Among all patients, 56% (N = 25,172) used oxygen at some point in their disease course. Overall, 37% of the cohort received oxygen prior to their diagnosis of IPF (N = 16,808), with the remainder initiating oxygen therapy either on or after their diagnosis of IPF (18.5%, N = 8,364). For those who initiated oxygen use prior to their IPF diagnosis, the average time between first oxygen use and IPF diagnosis was 814 days or 2.2 years (Fig. 2a). Those who initiated oxygen after their diagnosis of IPF, were begun on oxygen therapy an average of 283 days or less than 1 year after the diagnosis of IPF (Fig. 2b). Patient survival after oxygen initiation was an average of 3.03 (median: 2.47) years overall (Fig. 3a).

**Fig. 2a:**
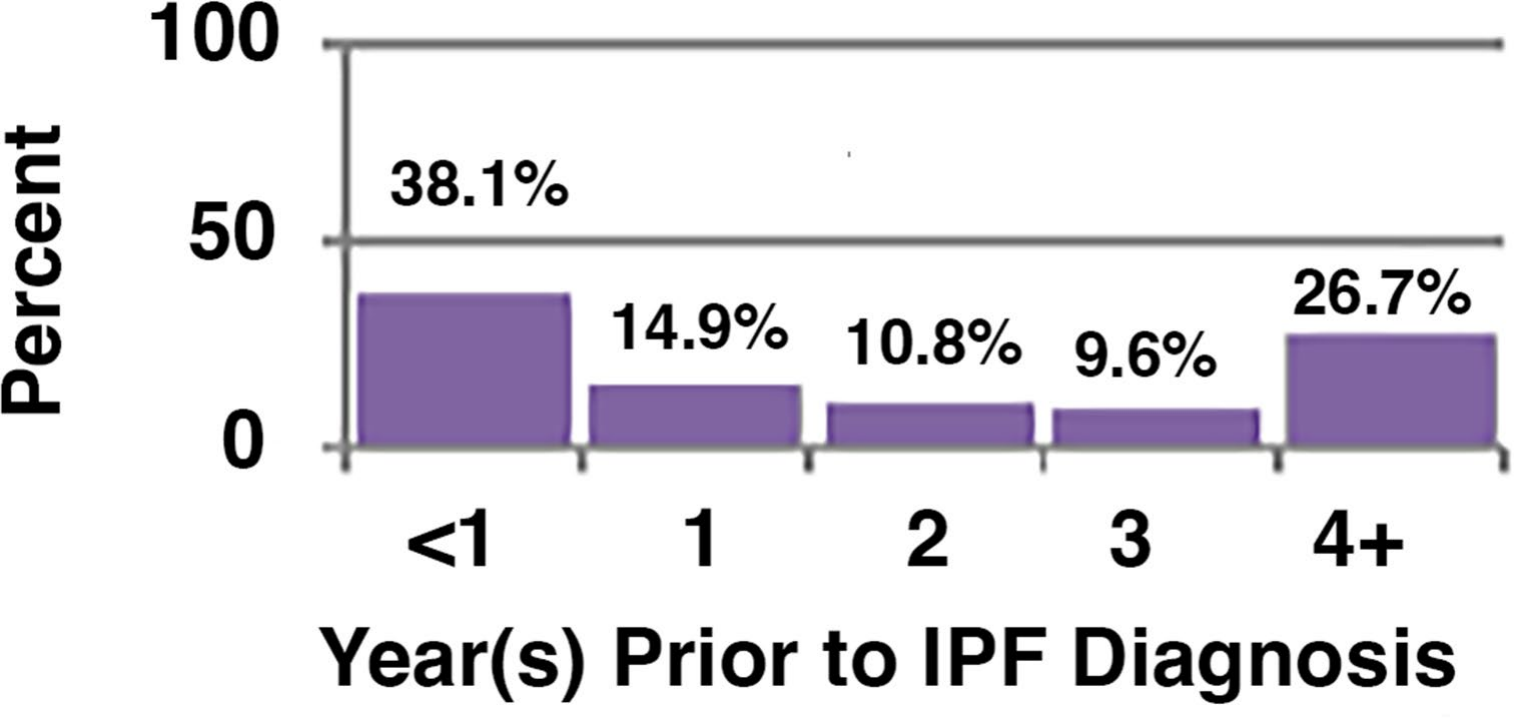
Timing of Oxygen Initiation, Prior to IPF Diagnosis, N = 16,808. (Timing of initiation of supplemental oxygen use before or after the index diagnosis of IPF. **A.** Relative timing of oxygen initiation in patients that were begun on oxygen therapy prior to the coded index diagnosis of IPF. Shown are the relative percentage of the total patients (N = 16,808) begun on oxygen therapy prior to the diagnosis of IPF, with the time of oxygen use expressed in years before the diagnosis of IPF).

**Fig. 2b:**
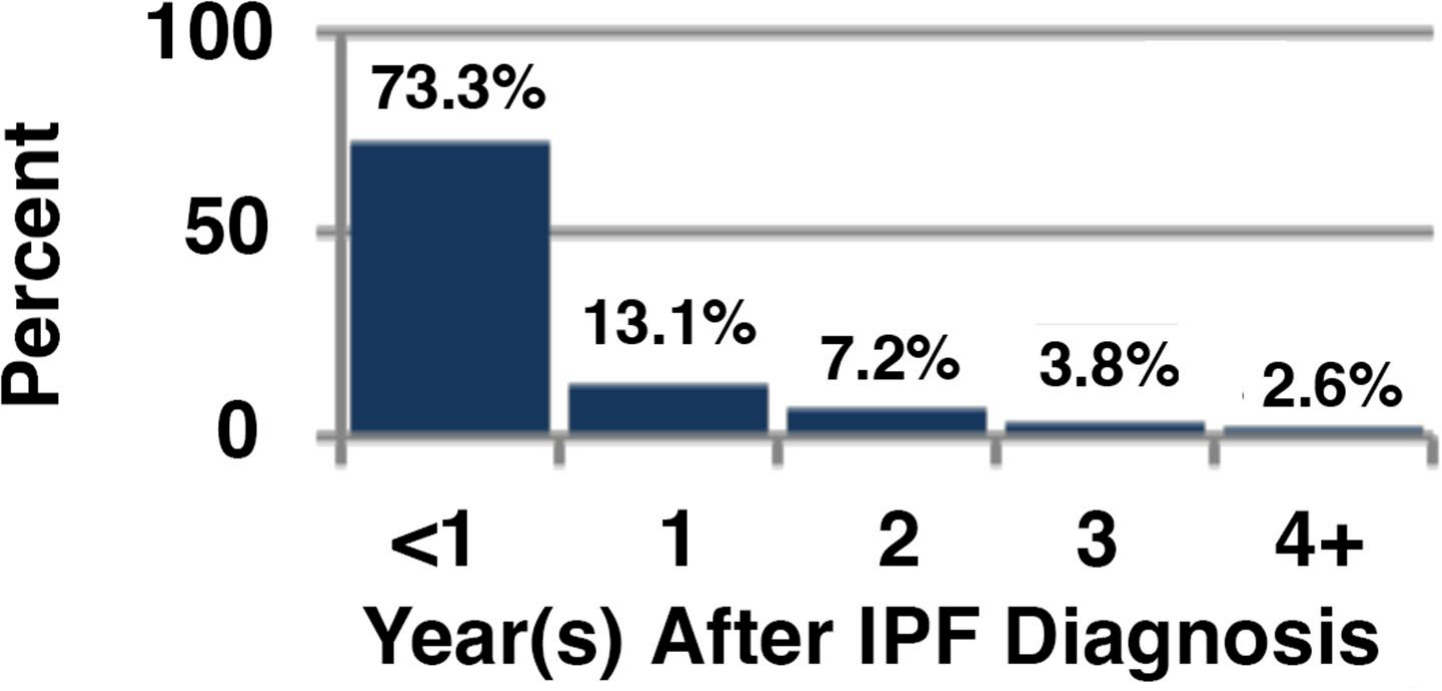
Timing of Oxygen Initiation, After IPF Diagnosis, N = 8,364. (Timing of initiation of supplemental oxygen use before or after the index diagnosis of IPF. **B.** Relative timing of oxygen initiation in patients that were begun on oxygen therapy after the coded index diagnosis of IPF. Shown are the relative percentage of the total patients (N = 8,364) begun on oxygen therapy after the diagnosis of IPF, with the time of oxygen use expressed in years after the diagnosis of IPF).

**Fig. 3a:**
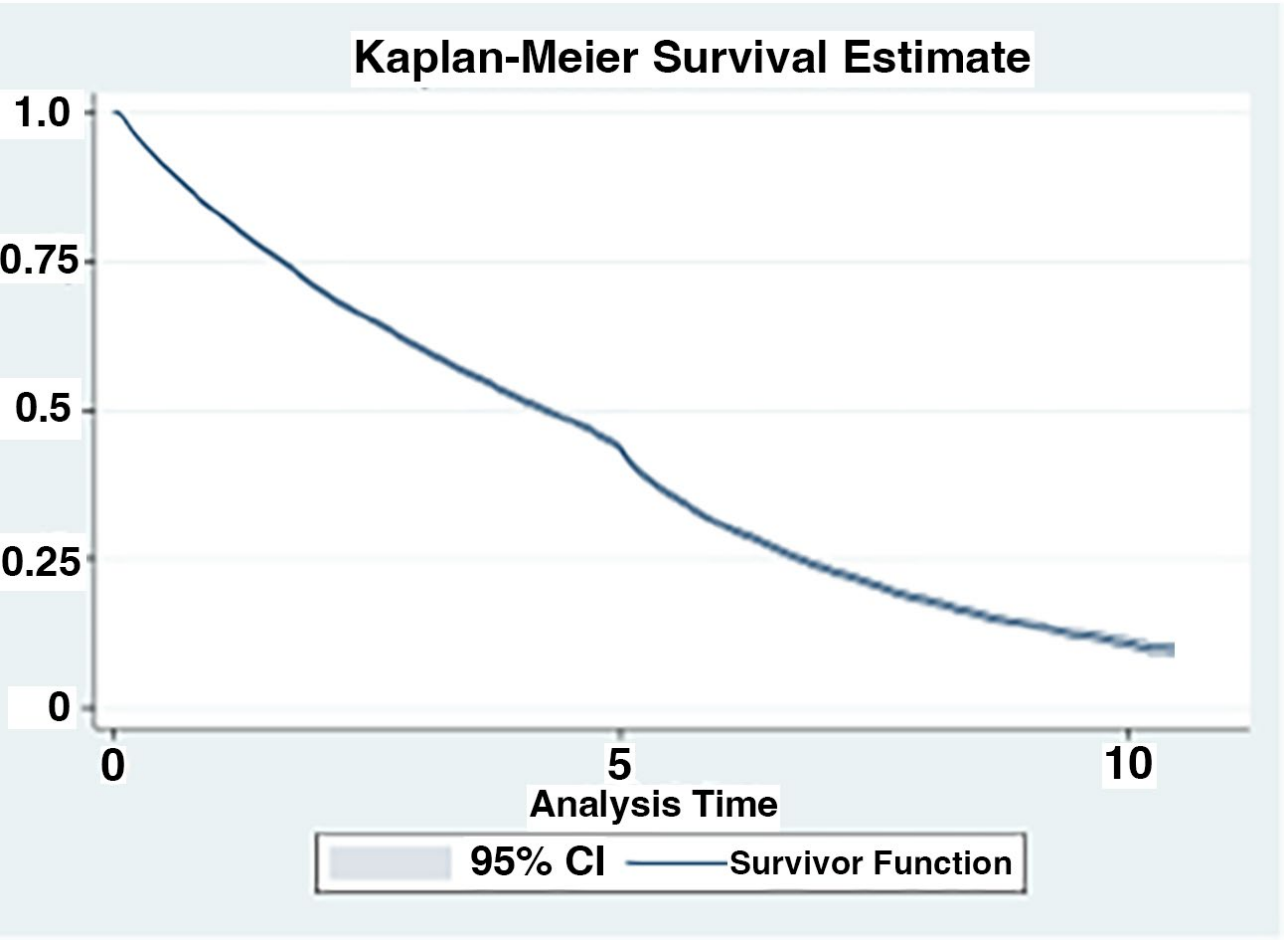
Survival Post-Initial Oxygen Use, N = 25,172. (Relative survival of patients with IPF in the cohort after initiation of oxygen or after the initial respiratory hospitalization. **A.** Shown is the Kaplan-Meier survival estimate of patients (N = 25,172) following initiation of oxygen therapy. The surviving fraction is expressed between 0 and 1.0, the time following initiation of oxygen therapy is expressed in years)

Survival declined drastically each year after respiratory hospitalizations, with an approximately 50% survival rate 2 years after the first respiratory related hospitalizations (Fig. 3b). The median survival time of this patient cohort overall was 2.80 years after the coded diagnosis of IPF. Also notable was that the lung transplantation rates were very low in this cohort. We found just 124 (0.28%) of individuals had received a transplant sometime after their IPF diagnosis. This may in part reflect the overall age of the individuals in the study population.

**Fig. 3b:**
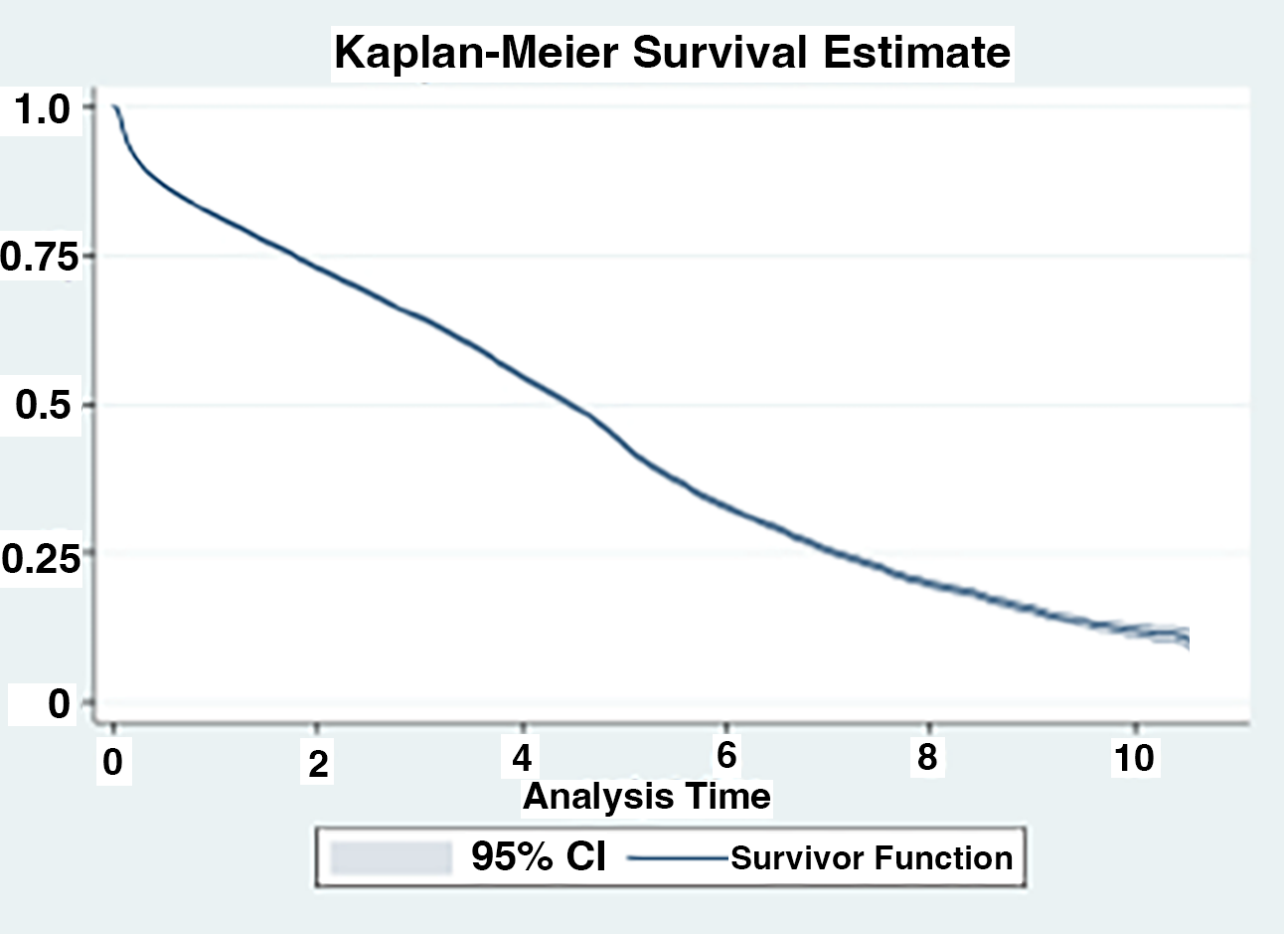
Survival Post-Initial Respiratory Hospitalization, N = 35,243. (Relative survival of patients with IPF in the cohort after initiation of oxygen or after the initial respiratory hospitalization. **B.** Shown is the Kaplan-Meier survival estimate of patients (N = 35,243) following initial respiratory hospitalization. The surviving fraction is expressed between 0 and 1.0, the time following initial respiratory hospitalization therapy is expressed in years)

## Discussion

It has been suggested that the diagnosis of IPF has been increasing in prevalence over time [16]. Despite the increasing prevalence and knowledge regarding this disease, patient prognosis remains poor [17]. However, most of our understanding of the diagnosis and prognosis of IPF is derived from single center series or registries based at academic centers. Our study describes a comprehensive overview of the diagnosis and treatment of IPF patients across the United States using a real-world claims-based dataset. This study provides a pre-diagnosis analysis and a broad overview of the time to diagnosis, time to treatment, and overall prognosis among patients with IPF in the U.S. While widely believed that patients with IPF experience significant diagnostic delays, this observational study provides insights among patients with IPF across all care settings. Unfortunately, our observations support the sobering reality of significant diagnostic delay of IPF and underutilization of available therapies for this disorder.

Our examined data (from 2014 to 2019) found a total number of patients with IPF = 44,891, yielding a prevalence estimate of 3 per 100,000 persons, per year. However, our cohort of patients with IPF (44,891) is certainly a significant underestimate of the total numbers with IPF. There are several reasons that indicate that the total of 44,891 patients in our cohort is an underestimate of the total numbers of patients with IPF. First, we required five years of consecutive enrollment and data to better understand the diagnostic timeline. In addition, we employed a comprehensive exclusion of competing diagnoses based upon our initial review of complete records on consecutive patients in our local electronic health records. Indeed, the available data initially searched yield over 90,00 patients with a code for IPF. However, our intent was to have a rigorously defined cohort of IPF with adequate timeline data to analyze diagnostic delays, treatment use, and survival. For these reasons, this estimate of IPF prevalence cannot be directly compared to other studies (which based prevalence on a single time point diagnosis to determine the number patients with this disorder).

Our data demonstrated that many patients received other respiratory diagnoses prior to their coded clinical diagnosis of IPF. The majority of these were pulmonary fibrosis unspecified, post-inflammatory fibrosis, chronic obstructive pulmonary disease unspecified, and interstitial pulmonary disease unspecified. It is also interesting that 19% of patients had three or more respiratory-related hospitalizations prior to diagnosis as well. Furthermore, roughly 37% of patients were already prescribed oxygen at the time their coded IPF diagnosis, with 27% of those receiving oxygen having been prescribed this therapy for 4 or more years prior to their diagnosis. These observations further support the conclusion that the diagnosis of IPF is rendered extremely late in the disease course.

It is concerning that only a small percentage of patients ultimately received anti-fibrotic treatment. Prior studies indicate that pirfenidone and nintedanib use is associated with slowing of lung function deterioration, as well as reduced short-term mortality and reduction in hospitalizations [4–6, 12]. In this large cohort, we observed that only 10% of patients were initiated on an anti-fibrotic medication, and a quarter of these patients did not refill their initial anti-fibrotic prescription. This could be due to many factors. It has been shown in previous studies that out-of-pocket costs are nearly 400 US dollars per month for each medication [4, 12]. Many patients also experience significant side effects when taking these medications [18]. Alternatively, given the late diagnosis of many of these patients, clinicians may view anti-fibrotic therapy as futile and may be hesitant to begin therapy in those with advanced disease.

Previous studies have also suggested gender differences in the initiation of anti-fibrotic therapy, with women less likely to receive anti-fibrotic therapy compared to male patients with IPF [4, 12]. In addition, there also appears to be ethnic and racial disparities between patients who are treated with anti-fibrotic agents. Our data demonstrated that, when compared to Caucasian patients, black, Hispanic, and other minorities were less likely to be initiated on anti-fibrotic medications [19]. This observation at least suggests possible differences in prescribing patterns. Such differences could potentially be related to socioeconomic factors including secondary costs of anti-fibrotic medications. Similar trends in prescribing disparities have been observed for other newer therapies, such as in the treatment of diabetes mellitus [20]. Additional studies to identify the root causes of the differences in diagnosis and treatment will be needed to develop strategies to address these disparities.

The prognosis for IPF from this real-world patient cohort was similar to other cohorts described, with a mean 2.88-year prognosis following clinical diagnosis [6]. This again supports our observation that diagnosis occurs late in the disease course. Early diagnosis provides the opportunity for early treatment initiation of the approved anti-fibrotic medications. It is important to note that there are several agents under investigation for patients with IPF. Unfortunately, studies of novel therapies often exclude patients with advanced disease. Hence, earlier diagnosis also provides the opportunity for participation in protocol-driven clinical trials. It does remain to be determined whether early diagnosis and implementation of such therapies will ultimately impact overall prognosis for patients with this deadly disease.

Our stated goal was to evaluate the timeline to diagnosis and therapy in patients with IPF. To accomplish this, we excluded patients with coexisting diagnostic codes for other fibrotic lung diseases including chronic hypersensitivity pneumonitis and connective tissue disease-associated lung fibrosis (Supplementary Figure S1). Interestingly, on our medical record review of consecutive patients identified using this coding strategy, many of these excluded patients fit within the category of pulmonary fibrosis with progressive phenotype. Such patients have been shown to benefit from anti-fibrotic treatment in a recent study [21]. Further analysis of these patience from this large cohort may provide additional understanding of the natural history of these diagnoses and their responses to therapy over time.

Additional studies using different methodologies have also documented delays in the diagnosis of interstitial lung disease and idiopathic pulmonary fibrosis. The INTENSITY study reported a survey of 600 subjects with diagnosed interstitial lung disease, finding that over half of patients had received at least one misdiagnosis, with delay to the current diagnosis occurring a median of seven months after the onset of symptoms and with 43% being delayed over a year [22]. Another report of patients with IPF from France, Germany, Japan and the United States, revealed diagnostic delays following the onset of symptoms ranging from 0.8 to 2.0 years [23]. Furthermore, a registry cohort of incident patients with IPF in Denmark reported a median diagnostic delay of 2.1 years [24]. Previous diagnoses before the diagnosis of IPF included heart disease, asthma, chronic bronchitis, and COPD [24]. Many of these studies are based on patients with incident IPF diagnosis from tertiary IPF centers or registries. Our study differs in that it surveys diagnostic delays and alternate diagnoses in a large claims-based data set in the United States using real world data across all care settings. As such, we report a somewhat longer delay in the diagnosis of IPF. Taken together with the other reports, concerns are raised that delay in the diagnosis of IPF are considerable, leading to late onset referral to specialty expertise and therapy. Of note, diagnostic delays greater than 1 year have been associated with worse progression-free survival [25].

### Limitations

This study does have several limitations. In a claims-based cohort dataset, we have no way to review the clinical notes and records to determine whether the medical team considered IPF earlier than when the code appeared in the claims-based record system. Furthermore, the respiratory related hospitalizations were determined using AHRQ software. We do not claim or report that the hospitalizations were solely due to IPF. They could have been coded as due to pneumonia, asthma exacerbation, post inflammatory fibrosis, COPD, or other diagnoses. It is not possible to retrospectively determine whether the hospitalizations were due to IPF alone from review of this claims-based data. That noted, we employed the greatest precision possible to determine the timing of diagnosis of IPF and related clinical events such as oxygen use, antifibrotics, hospitalizations and death.

In addition, as an observational retrospective dataset review, our findings rely on the validity of accurate recording and diagnostic coding and miscoding is a possibility which would impact our analysis. To mitigate this, we performed a local cohort code validation study to exclude concurrent competing codes to refine our study group to patients with IPF. Despite our concerted efforts to refine our study cohort, it remains possible that some patients with competing fibrotic diagnoses such as fibrotic NSIP, may have been included. Furthermore, as a descriptive survey study, no control group was required or reported and confounding factors could have impacted the results. We would also note that it also remains possible that some of prior respiratory diagnoses did represent simple infections rather than a true misdiagnosis of IPF. Finally, our analysis was limited to OptumLabs and Medicare Fee for Service data and may not be generalizable to patients that are uninsured or included under Medicaid based coverage. Our analysis was therefore limited to the United States and may not be generalizable to other parts of the world as access to healthcare and medication costs differ greatly across different geographic settings.

## Conclusions

Despite increasing knowledge regarding the risk factors and available treatments for IPF, delays in the diagnosis of this disorder and the initiation of therapy persist. Furthermore, prognosis remains poor following diagnosis. In addition, anti-fibrotic therapy is underutilized in our cohort. We hope to reduce the delay to IPF diagnosis and treatment initiation by increasing the awareness of care of IPF patients and by enhancing education for providers on the appropriate diagnostic approach.

## Electronic supplementary material

Below is the link to the electronic supplementary material.

Supplementary Material 1

## Data Availability

The data supporting the results of this study are third party data owned by OptumLabs and contain sensitive patient information; therefore, the data is only available upon request. Interested researchers engaged in HIPAA compliant research may contact connected@optum.com for data access requests. The data use requires researchers to pay for rights to use and access the data. General inquiries for data use can be submitted to the corresponding author, Dr. Andrew Limper, Gonda 18-South, Mayo Clinic, Rochester, MN 55,905; Tel.: (507) 284–4162; Fax: (507) 266–4372; E-mail address: limper.andrew@mayo.edu.

## List of abbreviations

IPF: Idiopathic pulmonary fibrosis
ILD: Interstitial Lung Disease
U.S.: United States
FDA: Federal Drug Administration
FVC: Forced vital capacity
6MWD: 6-minute walking distance
DLCO: Diffusing capacity of the lungs for carbon monoxide
OLDW: OptumLabs® Data Warehouse
ICD-9: International Classification of Diseases, Ninth Edition
ICD-10: International Classification of Diseases, Tenth Edition
HEDIS: Healthcare effectiveness data and information set
IPTW: Inverse probability treatment weighting
SD: Standard deviation
