# Supplementary material for: Idiopathic pulmonary fibrosis in the United States: time to diagnosis and treatment
Source: BMC Pulm Med. 2023 Aug 2;23:281. doi: 10.1186/s12890-023-02565-7 (PMC10398946; doi:10.1186/s12890-023-02565-7)
Supplement: Supplementary file 1 — Supplementary Material 1 [file 12890_2023_2565_MOESM1_ESM.docx]

**Supplementary Materials For**

**Idiopathic Pulmonary Fibrosis in the United States: Time to Diagnosis and Treatment.**

By Michelle B. Herberts, MD^1^ et al.

**Supplementary Figure S1.**

**Supplementary Figure S1. Distribution and relative frequency of ICD 9 and 10 Codes in the initial study database.** A retrospective cohort analysis was performed on patients 18 years of age and above that were evaluated at Mayo clinic between 2011 and 2019 and billed with ICD 9 and 10 codes for IPF (516.3 and J84.112). Once we identified these patients with these ICD codes, a cohort of physicians reviewed each patient chart to determine if they met the diagnostic criteria for IPF. Over 200 patients were reviewed. With the local cohort validation, it was determined that 69% of patients that were billed with these codes met the diagnostic criteria for IPF. Five percent met criteria for chronic hypersensitivity pneumonitis, 6% were diagnosed with connective tissue related ILD, 2% were diagnosed with RA related ILD, and 18% had other diagnoses. The most common disease in the “other” category included fibrotic non-specific interstitial pneumonia.

**Supplementary Table S1. Coded Diagnoses Excluded from the Final Study Cohort**

| **Disease** | **ICD–9** | **ICD–10** |
| --- | --- | --- |
| Post inflammatory pulmonary fibrosis | 515 | J84.110 |
| Chronic hypersensitivity pneumonitis | 495.5 | J67.9 |
| Rheumatoid arthritis | 446, 714, 720, 725, 701.0, 710.0, 710.1, 710.2, 710.3, 710.4, 710.8, 710.9, 711.2, 719.3, 728.5, 728.89, 729.30 | L94.0, L94.1, L94.3, M05, M06, M08, M12.0, M12.3, M30, M31.0, M31.1, M31.2, M31.3, M32, M33, M34, M35, M45, M46.1, M46.8, M46.9 |
| Sarcoidosis | 517.8 | I35 |
| Scleroderma | 710.1 | M34 |
| Mixed connective tissue disease | 710, 710.2, 710.3, 710.4 | M35.9 |
| Nonspecific interstitial pneumonia | 516.32 | J84.113 |

The following ICD-9 and ICD-10 diagnostic codes were identified and patients with these codes were excluded from the final study cohort
